# A tomato chloroplast-targeted DnaJ protein, SlDnaJ20 maintains the stability of photosystem I/II under chilling stress

**DOI:** 10.1080/15592324.2022.2139116

**Published:** 2022-11-21

**Authors:** Guohua Cai, Yujie Xu, Shuxia Zhang, Tingting Chen, Gan Liu, Zhengyue Li, Youshuang Zhu, Guodong Wang

**Affiliations:** School of Biological Sciences, Jining Medical University, Ri’zhao, 276800, P.R. China

**Keywords:** Chilling stress, DnaJ, photoinhibition, SlDnaJ20, tomato

## Abstract

DnaJ proteins are key molecular chaperones that act as a part of the stress response to stabilize plant proteins, thereby maintaining protein homeostasis under stressful conditions. Herein we used transgenic plants to explore the role of the tomato (*Solanum lycopersicum*) SlDnaJ20 chloroplast DnaJ protein in to the resistance of these proteins to cold. When chilled, transgenic plants exhibited superior cold resistance, with reduced growth inhibition and cellular damage and increased fresh mass and chlorophyll content relative to control. These transgenic plants further exhibited increased Fv/Fm, P700 oxidation, φ_Ro_, and δ_Ro_ relative to control plants under chilling conditions. Under these same cold conditions, these transgenic plants also exhibited higher levels of core proteins in the photosystem I (PSI) and II (PSII) complexes (PsaA and PsaB; D1 and D2) relative to control wild-type plants. Together these results suggested that the overexpression of *SlDnaJ20* is sufficient to maintain PSI and PSII complex stability and to alleviate associated photoinhibition of these complexes, thereby increasing transgenic plant resistance to cold stress.

## 1. Introduction

Cold stress is a common threat to a variety of terrestrial plants, with thermophilic vegetable crops including tomatoes (*Solanum lycopersicum*), cucumbers (*Cucumis sativus*), and bell peppers (*Capsicum annuum*) being particularly susceptible to chilling. Exposure to cold can impair plant growth and development, reducing both yield and quality. As photosynthesis is the key mechanism whereby plants accumulate biomass, this pathway is frequently targeted in an effort to improve crop yields.^1^ Importantly, owing to the complexity of the photosynthetic process it is very sensitive to cold stress.^2,3^

There are two primary stages to photosynthesis – light and dark reactions. During the light reaction stage, solar energy is converted into an electric signal, with electrons moving through the electron transport chain in the thylakoid membrane of chloroplasts, thereby facilitating the movement of H^+^ ions into the thylakoid cavity and generating a proton gradient that can be harnessed to mediate ATP synthesis during the dark reaction. The photosystem I (PSI) and photosystem II (PSII) complexes are key components of the electron transport chain, with the former being a multi-protein pigment complex that catalyzed electron transfer from plastocyanin (PC) to ferredoxin (Fd) in a series of steps.^4^ PSII is additionally a multi-pigment protein complex which facilitates the transfer of electrons from water molecules to plastoquinone (PQ) in the thylakoid membrane.^5^ Cold temperatures have been shown to destroy chloroplasts and to thereby adversely impact PSI and PSII stability, disrupting photosynthesis,^6^ leading to PSI and PSII photoinhibition.^4^ In the presence of light, the PSII D1 protein undergoes rapid turnover such that following the end of PSII photoinhibition this protein can be rapidly restored to mediate the recovery of photosynthetic activity.^4,7^ However, the PSI complex lacks any similar high turnover proteins, and as such following the termination of PSI photoinhibition many proteins must be synthesized de novo resulting in delayed recovery of photosynthetic activity.^8^

DnaJ proteins are key proteins that can act either on their own or in concert with Hsp70 proteins to mediate essential activities within cells such as protein folding, assembly, or degradation.^9–11^ Many studies have highlighted the importance of these DnaJ proteins for plant stress responses,^12,13^ with DnaJ proteins being expressed in nucleus, cytoplasm, endoplasmic reticulum (ER), mitochondria, and chloroplasts of cells.^14,15^ DnaJ proteins that localize to chloroplasts are well-characterized as being important mediators of plant stress responses.^16,17^ For example, the tobacco DnaJ protein Tsip1 is a zinc finger protein that is important for enhancing tobacco resistance to pathogenic or saline stress conditions.^18^ The AtJ8, AtJ11 and AtJ20 DnaJ proteins are all known to mediate photosynthetic pigment stability under high light conditions.^17^ The LeCDJ1 DnaJ protein is localized to chloroplasts and has been found to maintain PSII functionality under cold stress conditions.^19^ The SlCDJ2 protein localizes to chloroplasts and in response to heat stress serves to preserve Rubisco activity.^20^ While in *Chlamydomonas reinhardtii*, ZnJ6 is a thylakoid-associated DnaJ-like chaperone that assists in contributing to stress endurance, redox maintenance and photosynthetic balance.^21^

SlDnaJ20 is a DnaJ protein that localizes to chloroplasts in tomatoes, and that has been found to be upregulated in response to cold stress conditions.^22^ Herein we found that *SlDnaJ20* overexpression was associated with enhanced PSI and PSII complex stability, thereby reducing the photoinhibition of these complexes and enhancing transgenic tomato resistance to cold stress.

## 2. Materials and methods

### 2.1. Plant growth under cold stress conditions

We assessed the growth of seedlings of either wild type (WT) tomatoes (*S. lycopersicum* cv. L-402) or those the T_2_ generation of three different transgenic lines overexpressing S1DnaJ20 (OE2, OE6, and OE9). These seeds were initially planted in Murashige and Skoog medium, and were placed in a GPJ-400 (Dongpeng Instruments, Jiangshu, China) light incubator with a 16/8 hour light/dark cycle at 25°C, with a ~ 100 μmol·m^−2^·s^−1^ photon flux density (PFD). After 10 days, half of these seedlings were transferred to an incubator that was identical except for the temperature, which was set to 4°C, while the remaining seedling served as controls. Plants were imaged 5 days later, and control plants were then added to a quartz sand medium and transitioned to a greenhouse (25°C, 16 h light/dark, 55%–65% relative humidity, 200 μmol·m^−2^·s^−1^ PFD) where they were twice weekly administered Hoagland’s nutrient solution. After a further 4 week growth period, WT and transgenic plants were incubated at 4°C with ~100 μmol·m^−2^·s^−1^ PFD for 48 h and were then imaged.

### 2.2. Cell viability and physiological measurements

Cells were stained using Trypan blue as in previous studies.^23^ Both growth inhibition and cold resistance index values were derived based upon previously detailed approaches,^24^ while work by Kong et al was used to guide measurements of leaf chlorophyll content, relative electrical conductivity (REC), and malondialdehyde (MDA) levels.^17^

### 2.3. Assessment of Chlorophyll a fluorescence

A Handy PEA (Hansatech Instruments, Norfolk, UK) was used to measure chlorophyll *a* fluorescence as in previous studies,^25^ with PSII Fv/Fm calculated based upon the following: (Fm −Fo)/Fm.

### 2.4. Assessment of chlorophyll fluorescence

A FMS-2 (Hansatech, Cambridge, UK) was used to measure chlorophyll fluorescence as in previous studies,^17^ with qI defined as Fm/Fmr-1, and qE defined as Fm/Fm′-Fm/Fmr.

### 2.5. Western blotting

Proteins were extracted from the thylakoid membrane as in previous studies,^17^ with Western blotting conducted based on protocols published by Wang et al.^20^ PsaA, PsaB, D1, D2, and Actin (against 100 amino acids of recombinant actin conserved peptide) antibodies were purchased from Agrisera Company (Umea, Sweden).

### 2.6. Statistical analysis

SigmaPlot 12.5 (Systat Software, San Jose, CA, USA) and SPSS 18.0 (Chicago, IL, USA) were utilized for statistical testing, with data being means ± SD for three or more replicates. * p < .05 and ** p < .01 were the significance threshold.

## 3. Results

### 3.1 Overexpressing SlDnaJ20 improves tomato cold stress resistance

**Figure 1.**
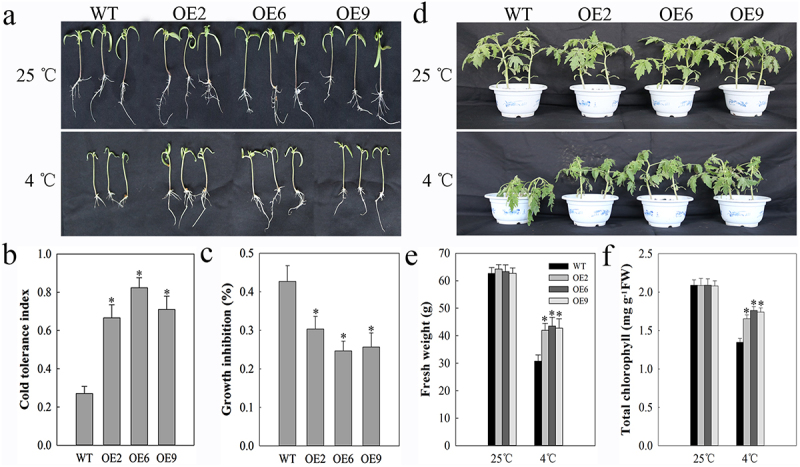
Cold resistance in 10-day-old and 6-week-old tomato plants. (a) 10-day-old seedlings were grown at either 25°C or 4°C for 5 d prior to imaging. (b) Cold resistance index. (c) Growth inhibition. (d) 6-week-old plants were grown at either 25°C or 4°C for 48 h. (e) Plant fresh weight. (f) Plant total chlorophyll content.

We have previously shown that chilling tomato plants to 4°C leads to the upregulation of *SlDnaJ20*.^22^ As such, we sought to observe the impact of this protein on tomato plant growth under cold stress conditions by observing the relative growth characteristics of WT and SlDnaJ20-overexpressing 10-day-old or 6-week-old plants in response to cold stress conditions. We found that at normal temperatures the WT and transgenic plants both grew normally with no significant differences in plant phenotypes or physiology (Figure 1). When plants were chilled for 4 days to 4°C, however, WT seedling growth inhibition was markedly more pronounced than was inhibition of the transgenic seedling growth (Figure 1a). We further confirmed seedling cold tolerance index values and growth inhibition rates (Figure 1, b and c). Similarly, after a 48 h cold stress period mature transgenic plants grew more readily than did WT controls (Figure 1d). Chlorophyll contents and fresh weight values were also significantly higher for transgenic plants relative to WT controls (Figure 1, e and f). As such, these results clearly demonstrated that overexpressing *SlDnaJ20* can significantly enhance tomato cold resistance.

### 3.2. Overexpression of SlDnaJ20 reduces cold-mediated damage to cell membranes

**Figure 2.**
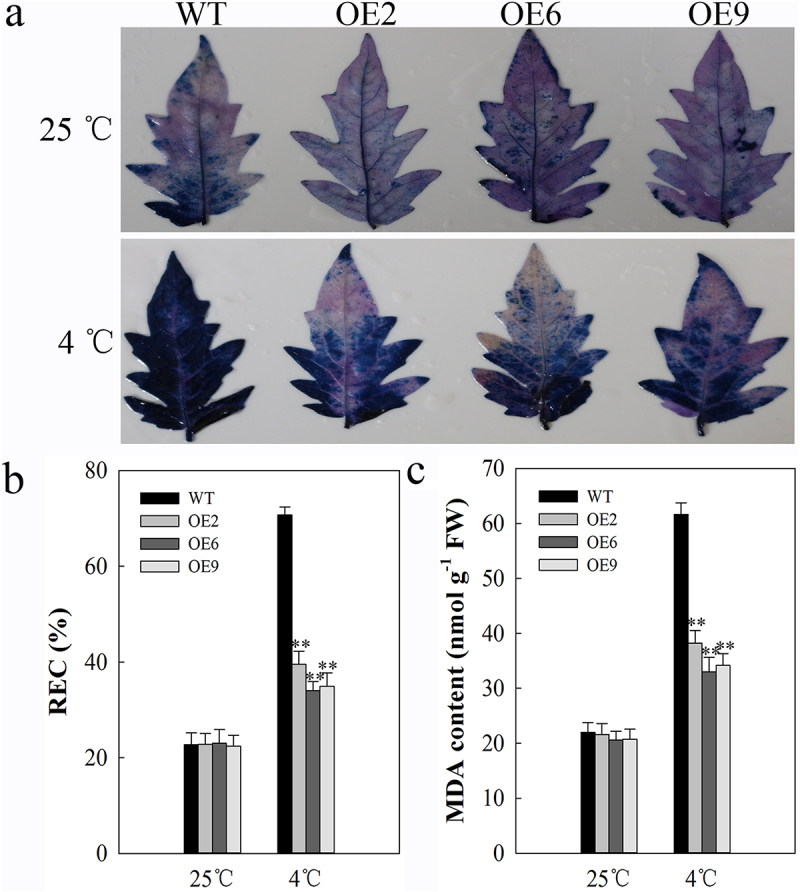
Measurement of cell damage in 6-week-old WT and transgenic plants. (a) Trypan blue staining, with plants incubated for 24 h at 25°C and 4°C shown in the upper and lower images, respectively. (b) REC. (c) MDA content.

Membrane damage is the primary site of cold-induced damage, with plant cell membranes transitioning from a liquid crystal state to a gel-like state. These changes markedly impair both membrane permeability and associated cellular functionality. To assess cell membrane changes in transgenic plants, we used Trypan blue staining which revealed similar staining when cells were grown at normal temperatures, but reduced blue staining for *SlDnaJ20*-overexpressing plants under cold conditions relative to WT controls consistent with reduced membrane damage (Figure 2a). We additionally analyzed the REC and MDA contents in cells under these same conditions as these two markers offer insight into the degree of cellular damage. As with the trypan blue staining, at control temperatures WT and transgenic plants exhibited comparable levels of these two markers, whereas at 4°C the levels of both REC and MDA rose, with increases being significantly higher in WT plants relative to transgenic plants (Figure 2, b and c). These findings thus revealed that elevated levels of *SlDnaJ20* can reduce cell membrane damage in response to cold stress.

### 3.3. Overexpressing SlDnaJ20 reduced PSI and PSII photoinhibition in response to cold stress

**Figure 3.**
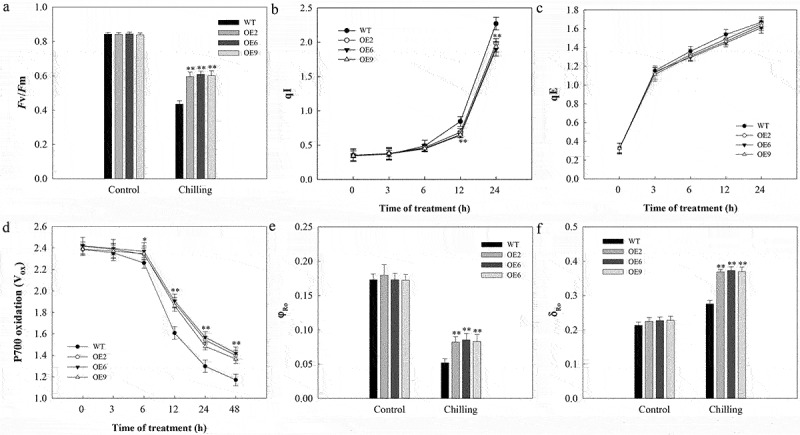
Assessment of PSI/PSII photoinhibition in 6-week-old WT and transgenic plants. (a) Fv/Fm. (b) qI. (c) qE. (d) P700 oxidation. (e) φ_Ro._ (f) δ_Ro._

The extent of PSII photoinhibition is commonly measured based upon maximal photochemical efficiency (Fv/Fm). When grown at normal temperatures, Fv/Fm values did not differ significantly between WT and transgenic plants, whereas these values declined in all plants in response to cold stress, with decreases in WT plants being significantly larger than those in transgenic plants (Figure 3a). This was consistent with SlDnaJ20 mediating reduced PSII photoinhibition in response to cold stress. There are multiple potential mechanisms whereby this protein may have reduced Fv/Fm values in this context, including changes in qI and qE values. We found that as the duration of cold exposure increased qI values in WT plants to a greater extent than in transgenic plants (Figure 3b). Elevated qI values are consistent with more substantial photoinhibition in WT plants under cold stress conditions relative to transgenic plants. In contrast, qE values did not differ significantly between these two plant groups, indicating that the elevated Fv/Fm values in transgenic plants may be a result of decreased qI values in these plants relative to WT controls (Figure 3c). We additionally analyzed three PSI activity-related fluorescent parameters (P700 oxidation, φ_Ro_ and δ_Ro_) in these plants and observed no differences in these values between WT and transgenic plants under normal growth conditions. In response to cold stress, however, transgenic plants exhibited less substantial reductions in P700 oxidation and φ_Ro_ relative to WT controls, with a correspondingly increased degree of δ_Ro_ elevation in these transgenic lines (Figure 3d, e and f). This thus suggested that overexpressing *SlDnaJ20* can reduce cold stress-induced PSI and PSII photoinhibition.

### 3.4. Overexpressing SlDnaJ20 reduces cold-induced PSI/PSII complex damage

**Figure 4.**
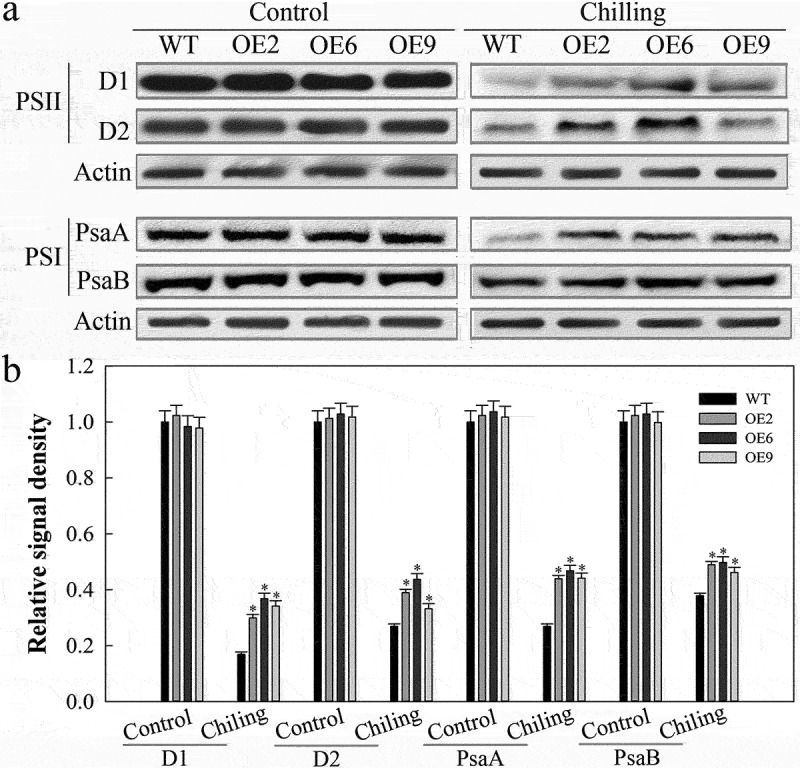
Western blot analysis of PSI and PSII core proteins. (a) PSI and PSII core protein levels in 6-week-old plants incubated for 48 h at 25°C and 4°C as assessed via Western blotting. (b) Quantitative image analysis of protein content in (a) using a Tanon Digital Gel Imaging Analysis System.

Finally, we sought to explore the mechanistic basis for SlDnaJ20-mediated reductions in cold-induced PSI and PSII photoinhibition in tomato plants via analyzing levels of core proteins involved in these two complexes. Under normal temperatures, the core proteins in the PSI (PsaA and PsaB) and PSII (D1 and D2) complexes did not differ significantly between WT and transgenic plants (Figure 4). After a 24 h exposure to cold stress, however, transgenic plants exhibited significantly higher levels of PSI and PSII core complex proteins relative to WT controls, suggesting that overexpressing *SlDnaJ20* helps to better maintain functionality of the PSI/PSII complex in response to chilling.

## 4. Discussion

Plant DnaJ proteins are widely expressed chaperone proteins that mediate environmental stress responses.^26,27^ While proteins in this family have been identified in a wide range of plants including rice, maize, and wheat, DnaJ expression and functionality in tomatoes are less well understood, particularly in the context of cold stress. We had previously found that incubation of tomato plants at 4°C led to induction of *S1DnaJ20*.^22^ As such, we explored the functional relevance of this DnaJ protein in tomato cold stress responses in both seedlings and mature plants, revealing that overexpressing *SlDnaJ20* enhanced transgenic tomato cold resistance significantly (Figure 1).

Cold stress can adversely impact many stages of plant growth, including germination, photosynthesis, yield, and quality. Extreme or prolonged cold stress can lead to significant plant death. In this study, we found that transgenic tomato plants exhibited reduced levels of cellular damage in response to cold stress than did WT controls, suggesting that overexpressing *SlDnaJ20* can protect tomato plants from cold-induced stress and associated damage (Figure 2). Cold conditions can impair photosynthesis, with inhibition of the PSI and PSII complexes being particularly pronounced upon chilling.^28,29^ We previously found S1DnaJ20 to localize to chloroplasts,^22^ consistent with our findings in the present study where in we observed significantly reductions in cold-induced decreases in PSII complex efficiently as measured based upon Fv/Fm in transgenic plants relative to WT controls (Figure 3a). Less substantial increases in qI values in transgenic plants were also consistent with this phenotype (Figure 3b), indicating that overexpressing *SlDnaJ20* reduced cold-induced PSII photoinhibition. With respect to PSI complex activity, we similarly found that transgenic plants exhibited less severe cold-induced decreases in P700 oxidation and φ_Ro_ values relative to WT controls (Figure 3, d and e), while cold-induced increases in δ_Ro_ were significantly larger than those in WT plants. These results thus revealed that overexpressing *SlDnaJ20* can relieve cold-induced PSI and PSII photoinhibition, suggesting a role for this protein in cold stress responses.

Cold stress can mediate the destruction of key components of the PSI and PSII complexes, thereby inducing profound photoinhibition.^3,30^ Both of these complexes are vital components of the photosynthetic electron transport chain, and are highly susceptible to cold stress.^6^ In the present report we found that overexpressing *SlDnaJ20* reduced cold-induced damage to both the PSI (PsaA and PsaB) and PSII (D1 and D2) complexes (Figure 4). We have previously shown SlDnaJ20 to interact with cpHSP70 in tomatoes.^19^ Together these findings thus suggest that SlDnaJ20 functions as a molecular chaperone to enhance protein folding, assembly, or stability under cold stress conditions.

In summary, overexpressing *SlDnaJ20* can effectively reduce the cold-induced photoinhibition of the PSI and PSII complexes via increasing their stability, thereby enhancing transgenic tomato resistance to cold stress.

## Abbreviations

Fd, ferredoxin; MDA, malondialdehyde; PC, plastocyanin; PFD, photon flux density; PSII, photosystem II; PSI, photosystem I; PQ, plastoquinone; REC, relative electrical conductivity; SlDnaJ20, *Solanum lycopersicum* DnaJ protein 20.

## References

[1] Kurek I, Chang TK, Bertain SM, Madrigal A, Liu L, Lassner MW, Zhu GH. Enhanced thermostability of *Arabidopsis* rubisco Activase improves photosynthesis and growth rates under moderate heat stress. Plant Cell. 2007;19:3230–6. doi:10.1105/tpc.107.054171. 17933901 PMC2174701

[2] Ensminger I, Busch F, Huner NPA. Photostasis and cold acclimation: sensing low temperature through photosynthesis. Physiol Plantarum. 2006;126:28–44. doi:10.1111/j.1399-3054.2006.00627.x.

[3] Fan JB, Hu ZR, Xie Y, Chan ZL, Chen K, Amombo E, Chen L, Fu JM. Alleviation of cold damage to photosystem II and metabolisms by melatonin in *Bermudagrass*. Front Plant Sci. 2015;6:925. doi:10.3389/fpls.2015.00925. 26579171 PMC4630300

[4] Huang W, Zhang SB, Cao KF. The different effects of chilling stress under moderate light intensity on photosystem II compared with photosystem I and subsequent recovery in tropical tree species. Photosynth Res. 2010;103:175–182. doi:10.1007/s11120-010-9539-7. 20221850

[5] Baena-González E, Barbato R, Aro EM. Role of phosphorylation in the repair cycle and oligomeric structure of photosystemII. Planta. 2013;208:196–204. doi:10.1007/s004250050550.

[6] Cai HM, Dong YY, Li YY, Li DX, Peng CY, Zhang ZZ, Wan XC. Physiological and cellular responses to fluoride stress in tea (*Camellia sinensis*) leaves. Acta Physiol Plant. 2016;38:144–155. doi:10.1007/s11738-016-2156-0.

[7] Jiang YP, Huang LF, Cheng F, Zhou YH, Xia XJ, Mao WH, Shi K, Yu JQ. Brassinosteroids accelerate recovery of photosynthetic apparatus from cold stress by balancing the electron partitioning, carboxylation and redox homeostasis in cucumber. Physiol Plantarum. 2013;148:133–145. doi:10.1111/j.1399-3054.2012.01696.x. 22998725

[8] Zhang S, Scheller HV. Photoinhibition of photosystem I at chilling temperature and subsequent recovery in *Arabidopsis thaliana*. Plant Cell Physiol. 2004;45:1595–1602. doi:10.1093/pcp/pch180. 15574835

[9] Hennessy F, Nicoll WS, Zimmermann R, Cheetham ME, Blatch GL. Not all J domains are created equal: implications for the specificity of Hsp40-Hsp70 interactions. Protein Sci. 2005;14:1697–1709. doi:10.1110/ps.051406805. 15987899 PMC2253343

[10] Craig EA, Huang P, Aron R, Andrew A. The diverse roles of J proteins, the obligate Hsp70 co-chaperone. Rev Physiol Biochem Pharmacol. 2006;156:1–21. doi:10.1007/s10254-005-0001-0. 16634144

[11] Xu HF, Dai GZ, Ye DM, Shang JL, Song WY, Shi HZ, Qiu BS. Dehydration-induced DnaK2 chaperone is involved in PSII repair of a desiccation-tolerant cyanobacterium. Plant Physiol. 2020;182:1991–2005. doi:10.1104/pp.19.01149. 32024697 PMC7140969

[12] Wang GD, Cai GH, Kong FY, Deng YS, Ma NN, Meng QW. Overexpression of tomato chloroplast-targeted DnaJ protein enhances tolerance to drought stress and resistance to *Pseudomonas solanacearum* in transgenic tobacco. Plant Physiol Bioch. 2014;82:95–104. doi:10.1016/j.plaphy.2014.05.011. 24929777

[13] Chen S, Qiu GL. Overexpression of seagrass DnaJ gene *ZjDjB1* enhances the thermotolerance of transgenic *Arabidopsis thaliana*. Physiol Mol Biol Plants. 2021;27(9):2043–2055. doi:10.1007/s12298-021-01063-6. 34629777 PMC8484434

[14] Miernyk JA. The J-domain proteins of *Arabidopsis thaliana*: an unexpectedly large and diverse family of chaperones. Cell Stress Chaperon. 2001;6:209–218. doi:10.1379/1466-1268(2001)006<0209:TJDPOA>2.0.CO;2. PMC434402 11599562

[15] Finka A, Mattoo RU, Goloubinoff P. Meta-analysis of heat- and chemically upregulated chaperone genes in plant and human cells. Cell Stress Chaperon. 2011;16:15–31. doi:10.1007/s12192-010-0216-8. PMC3024091 20694844

[16] Rajan VB, D’Silva P. *Arabidopsis thaliana* J-class heat shock proteins: cellular stress sensors. Funct Integr Genomic. 2009;9:433–446. doi:10.1007/s10142-009-0132-0. 19633874

[17] Kong FY, Deng YS, Zhou B, Wang GD, Wang Y, Meng QW. A chloroplast-targeted DnaJ protein contributes to maintenance of photosystem II under chilling stress. J Exp Bot. 2014;65:143–158. doi:10.1093/jxb/ert357. 24227338 PMC3883286

[18] Ham BK, Park JM, Lee SB, Kim MJ, Lee IJ, Kim KJ, Kwon CS, Paek KH. Tobacco Tsip1, a DnaJ-type Zn finger protein, is recruited to and potentiates Tsi1-mediated transcriptional activation. Plant Cell. 2006;18:2005–2020. doi:10.1105/tpc.106.043158. 16844903 PMC1533966

[19] Chen KN, Holmstrom M, Raksajit W, Suorsa M, Piippo M, Aro EM. Small chloroplast-targeted DnaJ proteins are involved in optimization of photosynthetic reactions in *Arabidopsis thaliana*. BMC Plant Biol. 2010;10:43. doi:10.1186/1471-2229-10-43. 20205940 PMC2844072

[20] Wang GD, Kong FY, Zhang S, Meng X, Wang Y, Meng QW. A tomato chloroplast-targeted DnaJ protein protects Rubisco activity under heat stress. J Exp Bot. 2015;66:3027–3040. doi:10.1093/jxb/erv102. 25801077

[21] Amiya R, Shapira M. ZnJ6 is a thylakoid membrane DnaJ-like chaperone with oxidizing activity in *Chlamydomonas reinhardtii*. Int J Mol Sci. 2021;22:1136. doi:10.3390/ijms22031136. 33498879 PMC7865324

[22] Wang GD, Cai GH, Xu N, Zhang LT, Sun XL, Guan J, Meng QW. Novel DnaJ protein facilitates thermotolerance of transgenic tomatoes. Int J Mol Sci. 2019;20:367. doi:10.3390/ijms20020367. 30654548 PMC6359579

[23] Choi HW, Kim YJ, Lee SC, Hong JK, Hwang BK. Hydrogen peroxide generation by the pepper extracellular peroxidase CaPO_2_ activates local and systemic cell death and defense response to bacterial pathogens. Plant Physiol. 2007;145:890–904. doi:10.1104/pp.107.103325. 17905862 PMC2048806

[24] Wang GD, Liu Q, Shang XT, Chen C, Xu N, Guan J, Meng QW. Overexpression of transcription factor SlNAC35 enhances the chilling tolerance of transgenic tomato. Biol Plant. 2018;62:479–488. doi:10.1007/s10535-018-0770-y.

[25] Zhang LT, Zhang ZS, Gao HY, Meng XL, Yang C, Liu JG, Meng QW. The mitochondrial alternative oxidase pathway protects the photosynthetic apparatus against photodamage in Rumex K-1 leaves. BMC Plant Biol. 2012;12:1471–2229. doi:10.1186/1471-2229-12-40. PMC3355048 22429403

[26] Xia Z, Zhang X, Li J, Su X, Liu J. Overexpression of a tobacco J-domain protein enhances drought tolerance in transgenic *Arabidopsis*. Plant Physiol Biochem. 2014;83:100–106. doi:10.1016/j.plaphy.2014.07.023. 25128645

[27] Salasmuñoz S, Rodríguez-Hernández AA, Ortega-Amaro MA, Salazar-Badillo FB, Jiménez-Bremont JF. *Arabidopsis* AtDjA3 null mutant shows increased sensitivity to abscisic acid, salt, and osmotic stress in germination and post-germination stage. Front Plant Sci. 2016;7:220. doi:10.3389/fpls.2016.00220. 26941772 PMC4766394

[28] Suzuki K, Nagasuga K, Okada M. The chilling injury induced by high root temperature in the leaves of rice seedlings. Plant Cell Physiol. 2008;49:433–442. doi:10.1093/pcp/pcn020. 18252732

[29] Zhang ZS, Yang C, Gao HY, Zhang LT, Fan XL, Liu MJ. The higher sensitivity of PSI to ROS results in lower chilling-light tolerance of photosystems in young leaves of cucumber. J Photoch Photo B. 2014;137:127–134. doi:10.1016/j.jphotobiol.2013.12.012. 24754967

[30] Dahal K, Kane K, Gadapati W, Webb E, Savitch LV, Singh J, Sharma P, Sarhan F, Longstaffe FJ, Grodzinski B, et al. The effects of phenotypic plasticity on photosynthetic performance in winter rye, winter wheat, and *Brassica napu*. Physiol Plant. 2012;144:169–188. doi:10.1111/j.1399-3054.2011.01513.x. 21883254

